# Immunostimulation and Immunoinhibition of Premalignant Lesions

**DOI:** 10.1186/1742-4682-4-6

**Published:** 2007-02-06

**Authors:** Richmond T Prehn

**Affiliations:** 1 Department of Pathology, University of Washington, 5433 South Hudson St, Seattle WA 98118, USA

## Abstract

**Background:**

The immune reaction may be either stimulatory or inhibitory to tumor growth, depending upon the local ratio of immune reactants to tumor cells.

**Hypothesis:**

A tumor-stimulatory immune response may be essential for survival of a neoplasm in vivo and for the biological progression from a premalignant lesion to a malignancy. Neither a positive nor a negative correlation between the magnitude of an immune-cell infiltrate and a cancer's prognosis can reveal whether the infiltrate was stimulating or inhibiting to the tumor's growth unless the position on the nonlinear curve that relates tumor growth to the magnitude of the immune reaction is known.

**Discussion:**

This hypothesis is discussed in relation to the development of human malignant melanomas and colorectal cancers.

## Background

**Figure 1:**
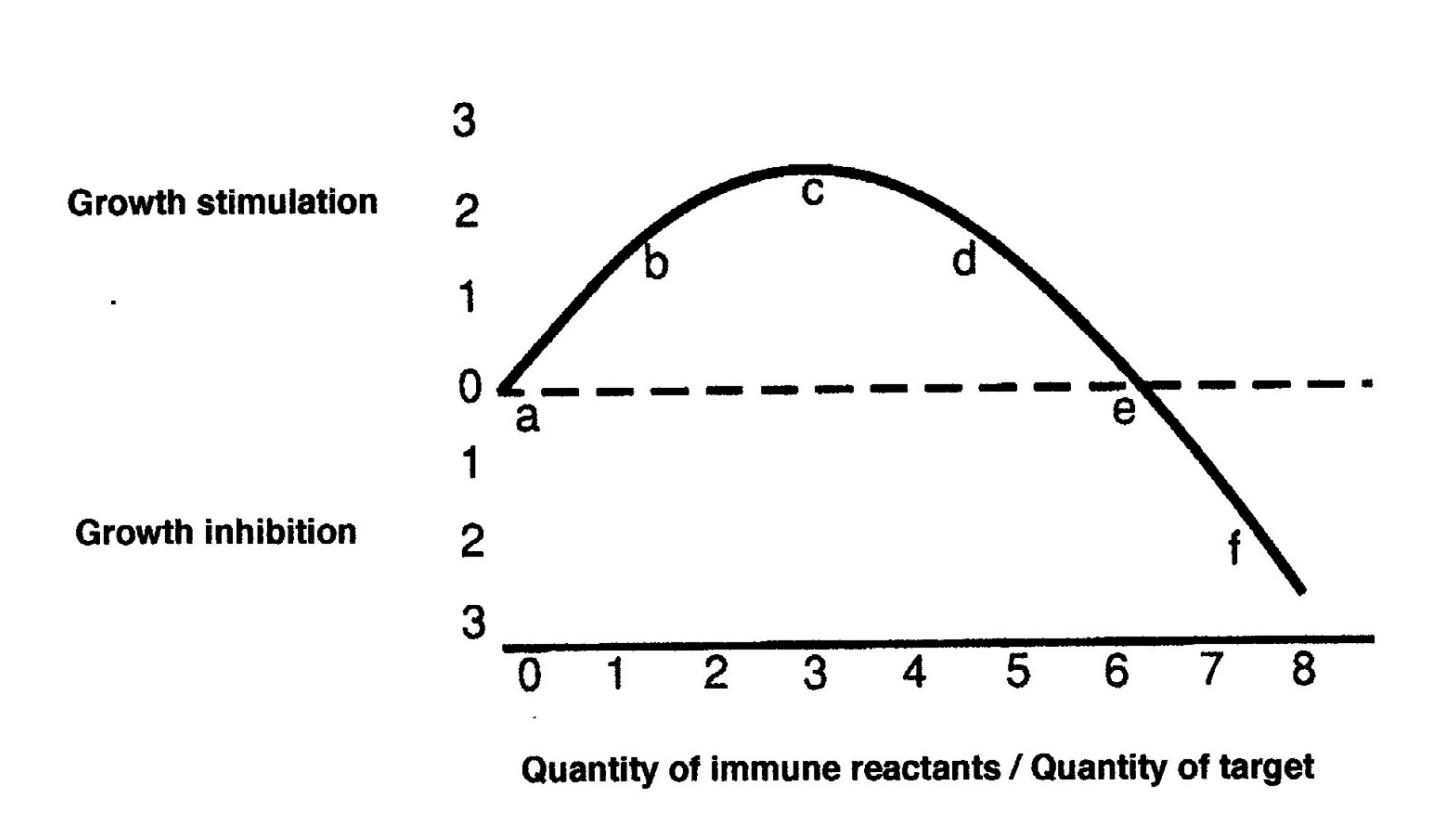
An idealized immune response curve [IRC] derived from data in [2]. The lettered and numbered points are arbitrary and designed only to facilitate the discussion.

The dose-response curve (ICR) relating the magnitude of the immune reaction to tumor growth is not linear, at least in the mouse (Figure 1). The fact that an immune reaction may, under some circumstances, act to enhance rather than inhibit neoplastic growth has been known for many years [1]. The first convincing demonstration that more might be involved than a mere blockage of a defensive immunity was probably a study with MCA-induced mouse sarcomas in a totally syngeneic system [2]. When various numbers of specifically immune spleen cells were mixed with a fixed number of tumor cells, the growths of the mixtures, when implanted into radiated and thymectomized syngeneic recipients, showed that the spleen cells were, relative to the effect of normal spleen cells, either stimulatory or inhibitory to the tumor's growth. Which result occurred *depended upon the local ratio of immune reactants to tumor cells*; *low ratios stimulated, but high ratios were inhibitory *[2]. A suggestively similar relationship was seen in vitro [3]. Thus, there is a problem in the interpretation of lymphatic infiltrates, especially in premalignant lesions; when is a lymphatic infiltrate stimulatory and when is it inhibitory to tumor growth?

In carcinogenesis, as with implanted tumors, whether stimulation or inhibition occurs probably depends upon where on the immune response curve (Figure 1) the system may be located. This location, in turn, would depend upon the intrinsic immune-capacity of the host and the immunogenicity of the tumor, perhaps as modified by trauma and/or inflammation [4]. It seems probable that an early lesion might be stimulated to grow by a weak incipient immune reaction, but later, as the immune reaction grew in magnitude, the effect might become inhibitory; if immunodepression moved the reaction from "d" to "b" on the curve in Figure 1, no net effect would be seen. Perhaps this is why Stutman, in his exhaustive review, found little evidence for either immunostimulation or immunoinhibition of carcinogenesis [5]. Furthermore, as I will subsequently explain, *a correlation between the density of an immune-cell infiltrate and the prognosis cannot indicate whether the infiltrate is helping or hindering the growth of the tumor*.

By "immune reaction" I refer to the algebraic sum of all those interacting parts that partake in the complex response to an antigenic stimulus: antibody, T cells, Tregs, NK cells, macrophages *etc*.; for a fuller discussion see [6,7]. While this essay is concerned with dosage effects, the quality of the immune reactants is also critical as the following quote from Kim *et al*. illustrates: "selective loss of Smad4-dependent signalling in T cells leads to spontaneous epithelial cancers throughout the gastrointestinal tract in mice, whereas epithelial-specific deletion of the Smad4 gene does not. Tumours arising within the colon, rectum, duodenum, stomach and oral cavity are stroma-rich with dense plasma cell infiltrates. Smad4(-/-) T cells produce abundant T(H)2-type cytokines including interleukin (IL)-5, IL-6 and IL-13, known mediators of plasma cell and stromal expansion" [8].

### Premalignant lesions

It is thought that most epithelial malignancies (and possibly all malignancies) arise in preexisting benign lesions. Among familiar examples are the occurrence of methylcholanthrene (MCA)-induced mouse-skin carcinomas in previously induced papillomas, the occurrence of human colorectal-carcinomas in preexisting colonic lesions, and the occurrence of human malignant-melanomas in preexisting nevi. The preexisting benign-lesions often undergo regression and rarely exhibit a malignant transformation; apparently, most benign lesions do not progress to malignancy.

### MCA-induced skin papillomas

Lappé showed that the incidence and the rate of regression of MCA-induced, mouse skin-papillomas can be affected by either increasing or decreasing the immune capacity of the host mice; a greater immune-capacity led to fewer papillomas, to fewer progressions to malignancy, and to earlier regressions of the papillomas; the converse effect was produced by lowering the immune capacity [9,10]. Lappé's method of producing the papillomas was to treat the skin of a normal mouse with a sub-carcinogenic dosage of MCA and then graft that skin onto a syngeneic mouse whose immunologic capacity had been raised or lowered by various techniques. The trauma of transplantation served as a "promoter" of the "initiated" skin.

In Lappé's system, the transformation rate of papilloma cells to malignancy was dependent upon the number and duration of papillomas and not upon a papilloma's degree of antigenicity. In other words, the transformation rate *per *papilloma-days at risk, was a constant and apparently independent of the immune response to individual papillomas [10].

It must be noted that in view of the nonlinear immune-response-curve, Lappé's results, when he decreased the immune response, could be explained equally well as a decreased immunoinhibition or as an increased immunostimulation; the decreased immune-reaction could have moved the system from near "f" toward "e", thus decreasing the tumor inhibition (see Figure 1). Alternatively, the decreased immune-reaction could have caused increased immunostimulation by moving the reaction from near "d" toward "c". I believe it will always remain uncertain whether one is dealing with changes in immunostimulation or immunoinhibition if the location on the dose-response-curve is unknown.

Andrews reinvestigated the mouse papilloma system using a modified technique; instead of transplanting the MCA-treated skin to isogeneic recipients, he used allogeneic. The allogeneic hosts had been maximally immunodepressed by radiation, thymectomy, and weekly administration of antithymocyte serum so that the skin grafts were not rejected. With the same standard dosage of MCA, most grafts developed papillomas, but about 80% of the papillomas regressed and *none progressed to carcinoma*. While there may have been some residual immune-capacity, that capacity was undetectable by several tests and, furthermore, the skin grafts, despite a major H-2 incompatibility, remained intact. It is possible that any residual immune-capacity was diverted from the papillomas to the normal allograft-tissue or was attenuated by a graft *versus *host reaction. In any event, Andrews concluded that papilloma regression could occur in the effective absence of an immunologic mechanism [11].

In essence, the work of Lappé suggests that papilloma incidence and papilloma regression have an immunologic basis, a finding consistent with both the immunosurveillance and the immunostimulation hypotheses, but the work of Andrews suggests that such a conclusion may not be the whole story; although immunity had been shown by Lappé to play a part, papillomas could nevertheless regress despite the apparent absence of immunity. Furthermore, although not commented upon by Andrews, *in the absence of immunity, the expected progression to malignancy was not observed *[11]. I have previously suggested, on other grounds, that an immune response might be *necessary *for carcinogenesis *in vivo *[7]. Furthermore, biological progression (dedifferentiation) within a neoplasm may be *aided *by an immune reaction [7,12].

In the work of Andrews, despite the lack of evidence of surviving immune-capacity, papillomas still appeared and still regressed [11]. This suggests that the papillomas were "promoted" primarily by the wound healing associated with skin grafting rather than by any residual immune-reaction. Subsequent papilloma-regression might have been caused by any number of possible non-immunologic mechanisms such as reaching a Hayflick limit or the lack of lymphotrophic support (there is much evidence that the lymphoid system can, with some degree of specificity, support the growth and regeneration of nonmalignant tissues and organs [13]).

### Human Melanoma

Let us now try to apply these ideas to a consideration of the biology of human melanoma. There seems to be a consensus that the incidence of malignant melanoma is increased in chronically immunodepressed kidney-transplant patients [14]. Many, and perhaps all, melanomas arise in benign nevi, many of which may, I hypothecate, be too small to be grossly visible; there is usually little or no discernible lymphoid infiltrate. Despite this absence of an infiltrate, the incidence of benign nevi is *much *increased in chronically immunodepressed kidney-transplant patients [15,16]; the incidence is also elevated in association with HIV infections suggesting that the increased incidence of nevi is probably caused by the immunosuppression *per se *[16]. It is possible, by analogy with the mouse skin papilloma system, that the increased incidence of malignant melanoma in immunosuppressed patients is entirely proportional to the increased number of nevi at risk for malignant transformation, but there are insufficient data to be certain.

At the time when a junctional nevus undergoes the rare transformation to melanoma there is, quite characteristically in people with normal immune capacities, a heavy lymphoid infiltration in the lesion. Later lesions may show less infiltrate, especially in metastases [17]. It has been reported that the density of the immune-cell infiltrate, in the surgical specimens from later lesions, correlates with survival [18], but this observation needs further substantiation. If true, one would be tempted by this observation to conclude that the infiltrate acts to inhibit tumor-growth, but such a tempting conclusion might be wrong even if the reported correlation were correct. If the reactions to the tumor were, in actuality, on the slope to the right of "c" on the IRC, those with more infiltrate would have a better prognosis owing to a shift toward "e" and thus toward a lesser degree of tumor stimulation rather than toward an increased tumor-inhibition (figure 1).

It seems to me most probable that the number of benign nevi increases in immunodepressed patients owing to the increase in the immunostimulation that would presumably be provided by the weakened immune reaction. In other words, I assume, largely because of the paucity of infiltrate, that in immunonormal individuals the usual reaction to nevi is not in the inhibitory range on the IRC, but somewhere around "d" or "e"; immunodepression would then move the reaction to the left toward "c" and greater stimulation of tumor growth.

A possible alternative explanation for the occurrence of many benign nevi, skin tumors, and lymphomas in immunosuppressed patients is a compensatory hyperplasia of cells that have some immune capacity (*e.g*., melanocytes can present antigen [19]. While this alternative cannot be excluded, it does not seem to easily explain the mixing experiments upon which the idea of the non-linear, dose-response curve is largely based [2,3].

The immune reaction obviously increases around the time of transformation to malignancy, as judged by the increased infiltrate, but whether or not it increases sufficiently to become inhibitory rather than stimulatory to the tumor's growth cannot, I believe, be ascertained from the available data. Any correlation of prognosis with the density of the infiltrate, in later surgical specimens, would be accommodated whichever were the case.

### Human Colorectal Papillomas

Recently, very convincing data have been published showing that in colorectal cancer the prognosis is indeed foretold by the degree of the immune-cell infiltrate in the surgical specimen; the greater the infiltrate in the surgical specimen the better the survival of the patient [20]. In fact, the lymphoid infiltrate proved to be a better predictor of the prognosis than did classical histologic criteria! As in melanoma, it seems to me most likely that the premalignant colorectal lesions would have aroused only a weak and stimulatory reaction, perhaps near "c" or "d" on the IRC. This stimulatory reaction may or may not have increased into the tumor-inhibitory range in the later overt carcinomas; in either case there would be the same correlation between the density of the infiltrate and the prognosis; a better prognosis with a denser infiltrate could imply *either less tumor stimulation or greater tumor inhibition*.

There is some reason to question the hypothesis that the infiltrate actually inhibits colorectal cancer. *Rectal *cancer, as distinguished from *colon *cancer, apparently has a *much lower *than expected incidence in heart and kidney allograft-patients [21] and a high percentage of colorectal lesions occur in the rectum. At least in mice, the close correlation between the tumor incidence at various points along the large bowel and the GALT (gut associated lymphoid tissue) may be significant [22]. The large lymphoglandular complexes in the rectum as compared with elsewhere in the colon led Steindl to coin the term "rectal tonsil"[23]. This is consistent with the hypothesis that immunostimulation of cancers may be greater in the rectum than in the rest of the large bowel and that the markedly lower incidence of rectal cancer in the immunodepressed as compared with immunonormal patients may be caused by the loss of much of this tumor-stimulation; *ie*., immunodepression may move the usual reaction from near "c" toward "a" on the IRC (figure 1).

## Discussion

These considerations lead, I think, to a disturbing question: is it possible that the immune reaction to an autologous or syngeneic cancer is *seldom *truly tumor-inhibitory? Even in the classical case in which immunity is produced against the growth of a highly immunogenic MCA-induced mouse sarcoma [24], can one really be sure that the increased immune reaction did not move the reaction from "c" on the IRC to "e"; would the tumor fail to grow if the reaction were moved only from "c" to "e" and not further into the truly inhibitory range? Andrew's work (previously discussed [11]) suggests that a tumor might not grow if the reaction were near either "a" or "e"; at neither location would there be effective immunostimulation. It could be that lack of stimulation rather than immune inhibition predominates in many or even in most situations in which immunity is associated with failure of a tumor to grow. But do such considerations really have more than academic importance?

How to determine the position on the IRC of a given cancer-induced immune-reaction would seem to be a matter of considerable importance, but, as far as I can determine, suitable methodologies are yet to be developed.

The mechanism by which an immune reaction can stimulate tumor growth has also not been elucidated. However, it may be useful to mention my own current hypothesis. Rubin has recently reviewed the extensive literature showing that the phenotypic stability of cells is usually maintained, despite their myriad mutations, by the influence of surrounding cells [25]. Thus, I propose that an immune reaction may, when present in less than lethal quantity, interact with cell-surface antigens to liberate tumor-cell growth by interfering with the normal tumor-inhibiting interactions among the cells.

## Abbreviations

IRC = immune response curve; MCA = 3-methylcholanthrene
